# 

**Published:** 1858-05

**Authors:** 


### RECEIPTS ON SUBSCRIPTIONS,

*From April 15th to May 15th*, 1858.

### ILLINOIS.

| | | |
|---|---|---|
| Dr. S. O. Long, | vol 1, | $2 00 |
| " A. Berchleman, | " 1, | 2 00 |
| " T. Hoffman, | " 1, | 2 00 |
| " R. Isham, | " 1, | 2 00 |
| " Geo. B. Richardson, | " 1, | 2 00 |
| " B. F. Ross, | " 1, | 2 00 |
| " J. W. Spalding, | " 5, 6 & 1, | 6 00 |
| " Isaac Rice, | " 1, | 2 00 |
| " —— Bennett, | " 6, | 2 00 |
| " B. Haines. | " 1, | 2 00 |
| " A. L. Kimber, | " 1, | 1 00 |
| " M. Davis, | " 1, | 2 00 |
| " W. R. Fox, | " 1, | 2 00 |
| " H. Nance. | " 1. | 2 00 |

### IOWA.

| | | |
|---|---|---|
| Dr. E. Keith, | vol. 1, | $2 00 |
| " J. R. Jay, | " 1, | 2 00 |

### INDIANA.

| | | |
|---|---|---|
| Dr. L. D. Personnett. | vol. 1, | $2 00 |
| " W. L. May, | " 1, | 2 00 |
| " E. H. Sutton, | " 1, | 2 00 |
| " W. T. Green, | " 6, | 2 00 |
| " A. W. Dewey, | " 1, | 2 00 |
| " J. C. Helm, | " 1, | 2 00 |
| " A. W. Adair, | " 1, | 2 00 |
| " H. Duncan, | " 6, | 2 00 |

### WISCONSIN.

| | | |
|---|---|---|
| Dr. W. A. Gordon, | vol. 1, | $2 00 |
| " M , | " 1, | 2 00 |

